# miRNA Gene Promoters Are Frequent Targets of Aberrant DNA Methylation in Human Breast Cancer

**DOI:** 10.1371/journal.pone.0054398

**Published:** 2013-01-16

**Authors:** Lukas Vrba, José L. Muñoz-Rodríguez, Martha R. Stampfer, Bernard W. Futscher

**Affiliations:** 1 Arizona Cancer Center, The University of Arizona, Tucson, Arizona, United States of America; 2 Department of Pharmacology and Toxicology, College of Pharmacy, The University of Arizona, Tucson, Arizona, United States of America; 3 Life Sciences Division, Lawrence Berkeley National Laboratory, Berkeley, California, United States of America; 4 Institute of Plant Molecular Biology, Biology Centre ASCR v.v.i., Ceske Budejovice, Czech Republic; University of Louisville, United States of America

## Abstract

miRNAs are important regulators of gene expression that are frequently deregulated in cancer, with aberrant DNA methylation being an epigenetic mechanism involved in this process. We previously identified miRNA promoter regions active in normal mammary cell types and here we analyzed which of these promoters are targets of aberrant DNA methylation in human breast cancer cell lines and breast tumor specimens. Using 5-methylcytosine immunoprecipitation coupled to miRNA tiling microarray hybridization, we performed comprehensive evaluation of DNA methylation of miRNA gene promoters in breast cancer. We found almost one third (55/167) of miRNA promoters were targets for aberrant methylation in breast cancer cell lines. Breast tumor specimens displayed DNA methylation of majority of these miRNA promoters, indicating that these changes in DNA methylation might be clinically relevant. Aberrantly methylated miRNA promoters were, similar to protein coding genes, enriched for promoters targeted by polycomb in normal cells. Detailed analysis of selected miRNA promoters revealed decreased expression of miRNA linked to increased promoter methylation for mir-31, mir-130a, let-7a-3/let-7b, mir-155, mir-137 and mir-34b/mir-34c genes. The proportion of miRNA promoters we found aberrantly methylated in breast cancer is several fold larger than that observed for protein coding genes, indicating an important role of DNA methylation in miRNA deregulation in cancer.

## Introduction

MicroRNAs (miRNA) are short single-stranded RNA molecules that regulate gene expression at the posttranscriptional level by stimulating the degradation or inhibiting translation of target mRNAs [1]. According to current estimates there are over a thousand miRNAs expressed from over five hundred transcriptional units (miRNA genes) encoded in the human genome. These miRNAs participate in the regulation of about two thirds of human genes and are involved in the determination of cell identity [2], [3]. Non-coding miRNA genes appear to be regulated in a fashion similar to protein-coding genes, such as through the actions of sequence-selective transcription factors and epigenetic control mechanisms [4], [5], [6], [7], [8]. Disruption of these regulatory mechanisms (e.g. DNA methylation) can produce abnormal chromatin states and participate in disease pathogenesis.

Aberrant DNA methylation linked to silencing of individual miRNA genes has been found in many cancer types including breast cancer [4], [9], [10], [11], [12], [13], [14], [15], [16], [17]. Some of these miRNAs function as tumor suppressors [16], [17] and their down regulation due to aberrant DNA methylation is associated with increased malignancy or metastatic potential in breast cancer [4], [14], [16], [17]. These reports illustrate the importance of epigenetic deregulation of miRNA expression in carcinogenesis; however, to our knowledge, a comprehensive analysis of DNA methylation of miRNA genes in breast cancer has not been reported.

In this study we identified miRNA promoters that undergo aberrant DNA methylation in human breast cancer using 5-methylcytosine immunoprecipitation coupled to analysis by a custom designed miRNA tiling microarray. The active human mammary miRNA promoter locations were previously determined by H3K4me3 ChIP analysis and miRNA expression correlated with H3K4me3 status of the promoters [5]. A large majority of these miRNA promoters are unmethylated in both normal human mammary epithelial cells (HMEC) and normal human mammary fibroblasts (HMF) and only become methylated in cancer; however, some miRNA promoters show normal cell type specific (CTS) patterns of DNA methylation, which are linked to their cell type specific patterns of expression [5]. These CTS miRNA differentially methylated regions (DMR) are a unique set of miRNA genes, since they are epigenetically labile, showing distinct and dynamic epigenetic patterns during normal cell differentiation and development. Due to their cell type specific patterns of DNA methylation, they also represent miRNAs especially susceptible to false positive or false negative interpretations. For example, while the CTS miRNA DMR can be readily detected and analyzed in pure cell populations (e.g. *in vitro* cell lines, LCM samples), they are more difficult to analyze in heterogeneous tumor specimens since, in addition to tumor cells, these specimens generally contain significant and variable proportions of normal epithelial and mesenchymal cells [18]. We used this knowledge to help inform and hone our analysis and identification of miRNA targets of aberrant DNA methylation in breast cancer.

Results from this study show that miRNA promoters are widely targeted for aberrant DNA methylation in breast cancer. Almost one third (55/167) of miRNA promoters active in mammary cells displayed hypermethylated DMRs in analyzed breast cancer cell lines. This percentage greatly exceeds the percentage of protein coding genes targeted for aberrant DNA methylation in cancer [19], [20]. Integration of our data with publicly available datasets showed that miRNA promoters targeted by polycomb repression in normal cells are more frequently DNA hypermethylated in cancer than other miRNA promoters; similar to what is observed in protein coding genes. As miRNA genes are disproportionately polycomb-regulated compared to protein-coding genes [5], this may help to explain why miRNA are more frequently targeted for aberrant DNA methylation during breast carcinogenesis. Forty two of the 55 miRNA promoters targeted for aberrant DNA methylation belong to the non-CTS class of miRNA genes while 13 belong to the CTS class of miRNA genes. Owing to cell heterogeneity of breast tumor specimens, only non-CTS miRNA promoters could be assessed for aberrant DNA methylation in clinical samples in this study. A majority of the non-CTS DMRs (29/42) were also found to be aberrantly methylated in individual breast cancer specimens, while the analysis of breast cancer specimens did not reveal any additional aberrantly methylated miRNA promoters beyond those observed in the breast cancer cell lines. Correlations between aberrant DNA methylation and expression of miRNAs showed only repressive to neutral effects of DNA methylation, but no stimulatory effects. Overall, our results show that miRNA genes are frequent targets of aberrant DNA methylation in cancer; the resulting deregulation of miRNA levels likely contributes to human breast carcinogenesis.

## Results

We have previously identified the promoter regions of miRNA genes expressed in normal breast cells represented by HMEC and HMF [5], and in this study we determined which of these promoters are targets for aberrant DNA methylation in breast cancer. DNA methylation of miRNA promoters was analyzed in seven breast cancer cell lines and twelve breast tumor tissue specimens as well as in three cultured normal finite lifespan HMEC strains and five breast non-tumor tissue specimens, using 5-methylcytosine immunoprecipitation coupled to custom tiling microarray covering miRNA gene regions.

**Figure 1:**
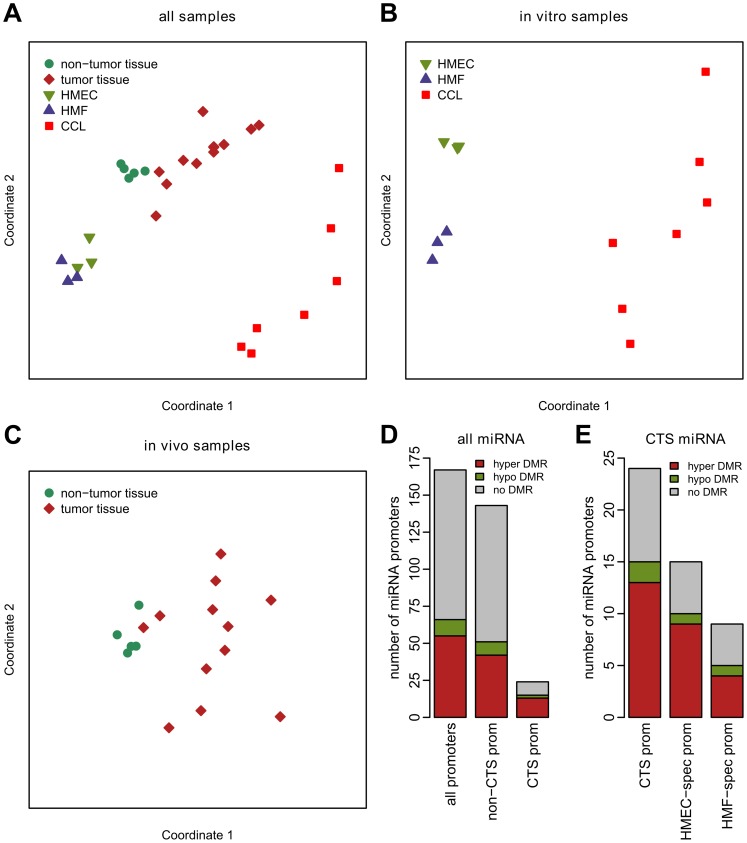
Microarray analysis of DNA methylation status of miRNA promoters. A, multidimensional scaling of pairwise distances derived from DNA methylation level of all miRNA promoter regions from all samples analyzed by microarray. CCL – cancer cell lines. B, same as A, but for *in vitro* grown cells only. C, same as A, but for tissue samples only. D, the proportion of miRNA promoters with DMRs in cancer cell lines. Displayed are data for all miRNA promoters, non-CTS promoters and CTS promoters. Hypermethylated promoters are in red, hypomethylated promoters in green and unchanged promoters in grey. E, the proportion of CTS miRNA promoters with DMRs in cancer cell lines. Displayed are data for all CTS miRNA promoters and those specific for either HMEC or HMF.

Multidimensional scaling analysis of DNA methylation microarray data from all miRNA promoters revealed a link between DNA methylation status of miRNA gene promoters and the phenotype of individual specimens (Figure 1A). In this analysis, the normal state was represented by cultured pure populations of finite lifespan HMEC and HMF, as well as non-tumor breast tissue from surgical specimens. Tumor samples were represented by pure populations of breast cancer cell lines as well as heterogeneous tumor tissues, containing a complex mixture of cell types. Figure 1A shows that normal *in vitro* and *in vivo* samples form two clusters on the left side of the multidimensional scaling plot, while the tumor samples are more dispersed. The most separated are the pure cancer cell lines and the pure normal HMEC and HMF strains. Probably due to their heterogeneous nature, the distance between tumor and non-tumor tissues is smaller, nevertheless the direction of the change is the same, indicating similar DNA methylation differences in both cancer cell lines and tumor tissue. To discern possible effects of *in vitro* culture versus heterogeneity of *in vivo* tissues we also analyzed the *in vitro* and *in vivo* samples separately (Figure 1B,C). Again there is overall larger difference between cultured cancer cell lines and HMEC or HMF cells than between tumor tissue and non-tumor tissue. Analysis of the *in vitro* samples alone also shows the physiological DNA methylation differences between HMEC and HMF (Figure 1B). Overall, these observations indicate that the DNA methylation status of a large portion of miRNA promoters is changed in cancer samples.

We next sought to determine which miRNA promoters are affected by changes in DNA methylation. First, we analyzed all promoters for the presence of DMRs in seven breast cancer cell lines relative to three genotypes of normal pre-stasis finite lifespan HMEC. Almost one third (55 of 167 or 33%) of miRNA promoters active in normal mammary cells became hypermethylated in cancer cells (Figure 1D), while only eleven promoters (6.6%) became hypomethylated. The full list of miRNA promoters with DMRs is included in Table 1 and Table S1.

**Table 1:** miRNA promoters with hypermethylated DMRs in breast cancer cell lines.

non cell-type specific:	HMEC specific:	HMF specific:
mir−335, mir−1305, mir−1307,	mir−205, mir−944,	mir−497/mir−195,
mir−106a/mir−18b/mir−20b/mir−19b−2/mir−92a−2/mir−363,	mir−200c/mir−141,	mir−490,
mir−1301, mir−1306, mir−874, mir−149, mir−455,	mir−187,	mir−199b,
mir−1179/mir−7−2, mir−548a−3, mir−142, mir−769,	mir−135b,	mir−10b
mir−196a−1, mir−941−1/mir−941−2/mir−941−3, mir−320c−2,	mir−582,	
mir−31, mir−196a−2, mir−130a, mir−30e/mir−30c−1,	mir−1910,	
mir−148a, let−7g, let−7a−3/let−7b, mir−708, mir−585,	mir−584, mir−577	
mir−298/mir−296, mir−129−2, mir−129−1, mir−153−1,		
mir−101−2, mir−101−1, mir−212/mir−132, mir−137,		
mir−34b/mir−34c, mir−196b, mir−99a/let−7c, mir−155,		
mir−615, mir−125b−1, mir−125b−2, mir−140, mir−1228		

We have previously shown that normal CTS miRNA expression is often regulated by epigenetic mechanisms including DNA methylation [5]. To determine whether the promoters of these CTS miRNAs are more likely to become methylated in cancer, we split the miRNA genes into cell-type specific and those without distinct cell-type specificity to determine the proportions of promoters with DMRs in individual groups of miRNA genes in cancer cells. Most of the promoters (143) belong to the non CTS miRNA genes; they contain hypermethylated DNA in 42 promoters (29%) (Figure 1D, Table 1). CTS miRNA promoters, although lower in number (24), have significantly higher (p-val = 0.017) proportion of hypermethylated promoters (13 out of 24 or 54%) (Figure 1D). When these 24 promoters are further split into HMEC and HMF specific, we found hypermethylation in 9 out of 15 (60%) HMEC specific and in 4 out of 9 (44%) HMF specific miRNA promoters, respectively (Figure 1E, Table 1). Overall, these data show that miRNA gene promoters are frequent targets of DNA hypermethylation in cancer. The hypermethylation is common for both CTS and non CTS miRNA genes, although the CTS miRNA genes are hypermethylated more frequently.

**Figure 2:**
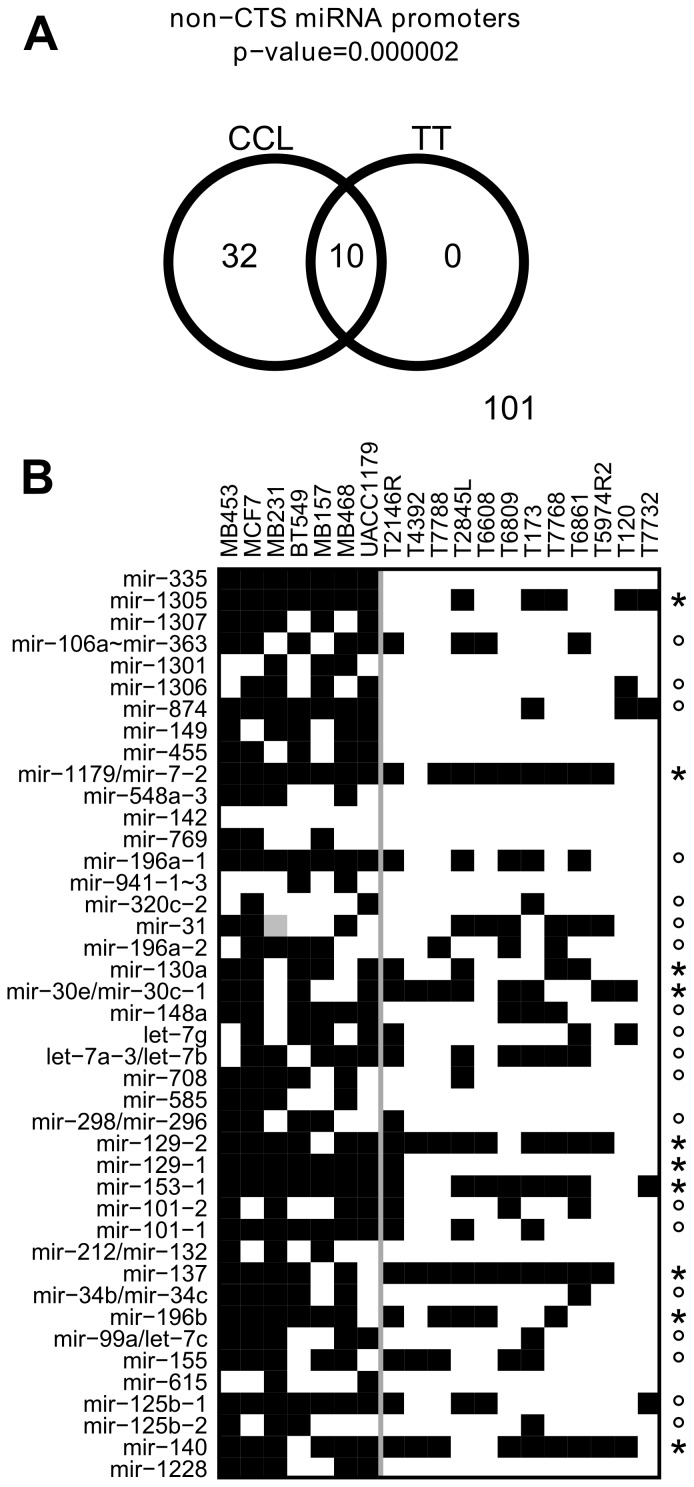
MicroRNA promoters hypermethylated in cancer cell lines are hypermethylated also in tumor tissue samples. A, Venn diagram showing the overlap between non CTS miRNA promoters hypermethylated in cancer cell lines (CCL) and those hypermethylated in tumor tissue (TT) samples. p-value of the significance of the overlap is shown at the top. B, Hypermethylation status for 42 non-CTS miRNA promoters significantly hypermethylated in cancer cell lines group. Shown are data for individual cancer cell lines and individual tumor tissue samples analyzed by microarray. miRNA genes are listed on the left, sample names are on the top. miRNA promoters that are significantly hypermethylated in tumor tissue samples as a group are marked by an asterisk on the right. Additional miRNA promoters that are significantly hypermethylated in at least one tumor specimen are marked by a circle sign on the right. Cancer cell lines and tumor tissue samples are separated by vertical grey line. The mir-31 promoter of MB231 sample is grey because this cell line has a homozygous deletion of this region. mir-106a∼mir-363 designates mir-106a/mir-18b/mir-20b/mir-19b-2/mir-92a-2/mir-363 cluster and mir-941-1∼3 designates mir-941-1/mir-941-2/mir-941-3 cluster.

We further sought to determine whether the changes in DNA methylation observed in the *in vitro* cultured cancer cell lines also occur *in vivo* in breast tumors and therefore may have clinical relevancy. To avoid possible confounding effects from different proportions of differentially methylated normal cell types (HMEC and HMF) in individual tissue samples, we also restricted this analysis to non-CTS miRNA promoters. Hypergeometric test revealed highly significant overlap between non-CTS miRNA promoters containing hypermethylated DMRs in cancer cell lines and those hypermethylated in a group of twelve tumor tissues (Figure 2A). For non-CTS miRNA, 10 out of 42 promoters hypermethylated in cancer cell lines also contained hypermethylated DMRs in tumor tissues group (Figure 2A). Furthermore, the tumor tissue did not show any significant DMRs not represented in the cancer cell lines group (Figure 2A), indicating that cancer cell lines represent most (if not all) of the DNA methylation changes observed in *in vivo* tumor tissues. Finally, 19 of 32 miRNA promoters that do have significant hypermethylated DMRs in the cancer cell lines group, but are not significantly hypermethylated in the tumor tissue group (Figure 2A left part), contain hypermethylated DMRs in at least one individual tumor sample (Figure 2B). The remaining 13 promoters that were found to be hypermethylated in cancer cell lines, but were not detected as DMR in the tumor tissue samples, were either not present in the analyzed cohort or were undetectable due to the tissue heterogeneity. Overall these data show that a majority of the miRNA promoters aberrantly methylated in breast cancer cell lines are also aberrantly methylated in *in vivo* breast cancer samples and may therefore be clinically relevant and not an artifact of tissue culture.

**Figure 3:**
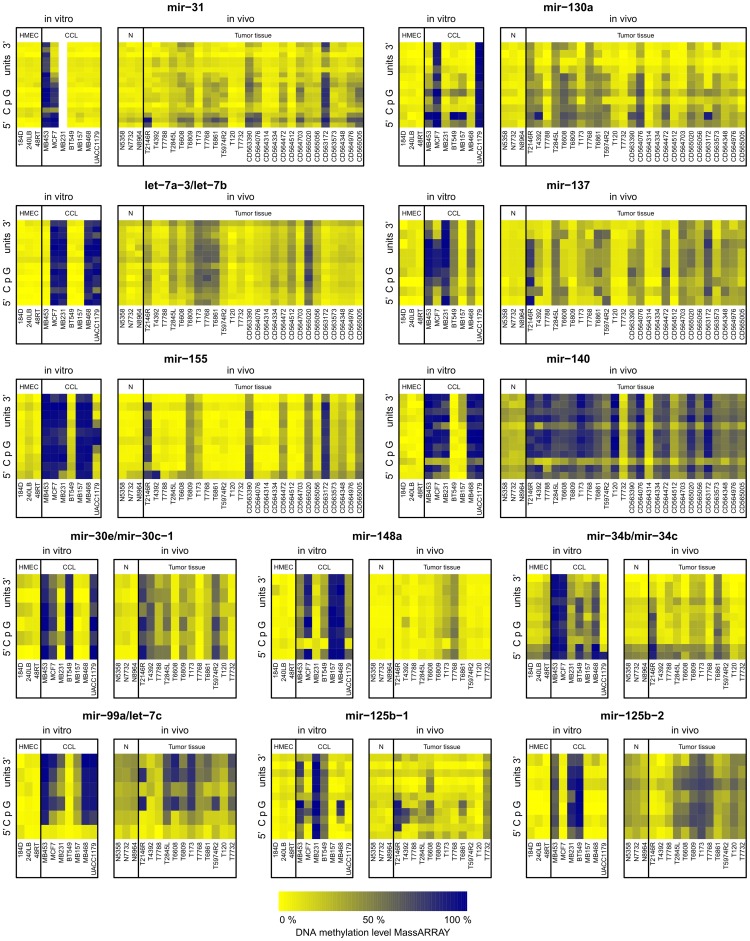
MassARRAY analysis of DNA methylation status of twelve selected miRNA promoters. MassARRAY amplicons 350–550 bp in length were targeted to the regions, where DMRs were present in most cancer samples accordinng to microarray analysis. The data are represented as heatmaps with low percentage of methylation in yellow and high percentage of methylation in blue. The data of each miRNA promoter is shown in two panels; one for pure *in vitro* grown cells and the other for heterogenous tissue samples. The sample groups are labeled at the top. The columns contain data for individual samples labeled at the bottom. The rows show data from individual CpG units for MassARRAY amplicons. The six miRNA promoters that were analyzed on an expanded tumor sample set are at the top part of the figure. The data for the mir-31 promoter of MB231 sample are missing because this cell line has a homozygous deletion of this region.

**Figure 4:**
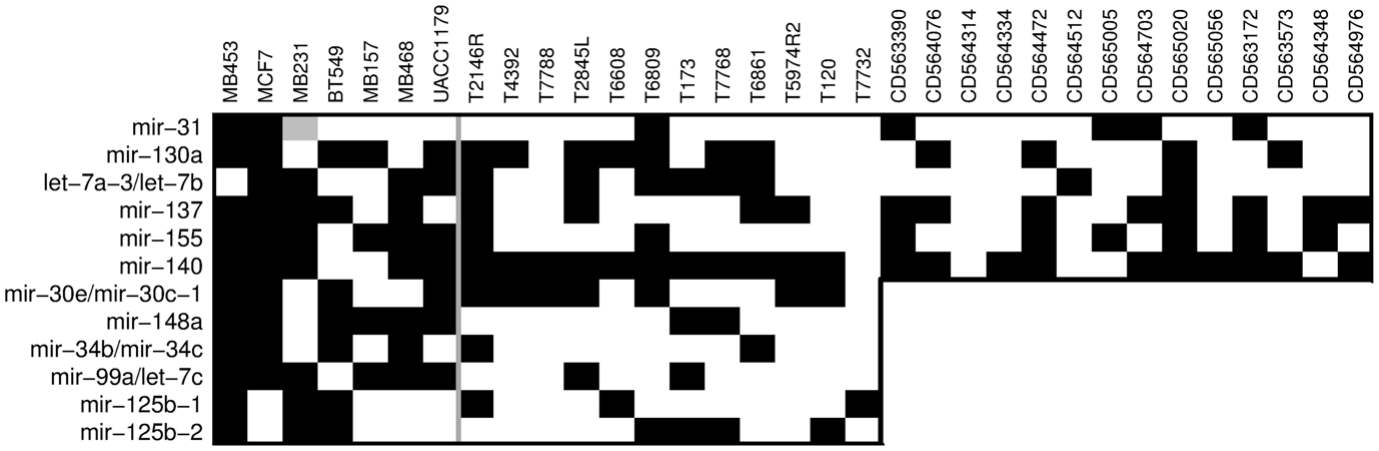
Overview of DNA hypermethylation status for 12 miRNA promoters analyzed by MassARRAY. miRNA genes are listed on the left, sample names are on the top. miRNA promoters that have significant ≥20% increase in DNA methylation within the whole MassARRAY amplicon relative to normal samples are in black. For a select set of miRNAs, 14 additional breast cancer specimens were analyzed. Cancer cell lines and tumor tissue samples are separated by vertical grey line. The mir-31 promoter of MB231 sample is grey because this cell line has a homozygous deletion of this region.

To verify and expand the microarray data, twelve of the differentially methylated miRNA promoters representing those with significant hypermethylation in the tumor tissue group (mir-130a, mir-30e/mir-30c-1, mir-137, and mir-140) as well as those hypermethylated in individual tumor tissue samples only (mir-31, let-7a-3/let-7b, mir-155, mir-148a, mir-34b/mir-34c, mir-99a/let-7c, mir-125b-1, and mir-125b-2) were analyzed by Sequenom MassARRAY (Figure 3). In addition to the samples analyzed by microarray, six of these promoters (mir-130a, mir-137, mir-140, mir-31, let-7a-3/let-7b, mir-155) were also analyzed on a larger sample set of 7 cancer cell lines and 26 breast tumor specimens (Figure 3 (top part)). The MassARRAY data in general confirmed the observations from DNA methylation microarray analysis (Figure 4). Similar increases in DNA methylation were also observed in the additional tumor tissue samples that were not part of the original microarray analysis (Figure 3 (top part), Figure 4 (top part)). The level of DNA methylation observed in the heterogeneous tissue samples was frequently lower than in the cancer cell lines (Figure 3), likely due to the cancer cells in tumor tissue being diluted by normal cells lacking the hypermethylation. This situation also makes the detection by microarray of hypermethylated regions in the tumor tissue more difficult and may help to explain the higher number of significant DMRs detected in pure cancer cell lines. Overall, these data confirm the findings from the microarray analysis, and show that these twelve hypermethylated DMRs observed at non-CTS miRNA promoters in *in vitro* grown cancer cell lines could also be found in *in vivo* tumor tissue samples, including additional samples independent of the original tumor cohort.

**Figure 5:**
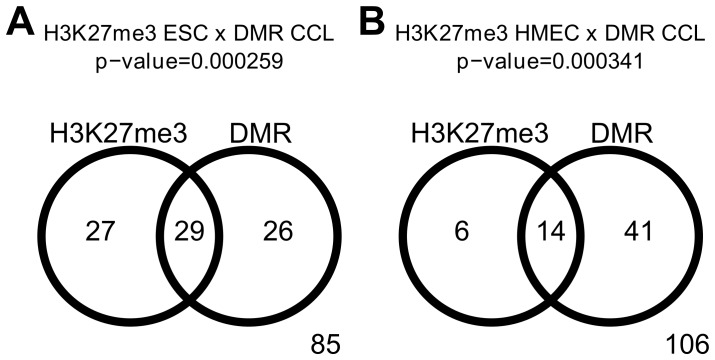
MicroRNA promoters occupied by polycomb specific H3K27me3 in normal cells are frequently hypermethylated in cancer. A, Venn diagram showing the overlap between promoters occupied by H3K27me3 in ESC and those hypermethylated in breast cancer cell lines (CCL). B, Venn diagram showing the overlap between promoters occupied by H3K27me3 in HMEC and those hypermethylated in breast CCL. Both overlaps are highly significant (hypergeometric test).

Polycomb targeted genes are frequent targets of aberrant DNA methylation in cancer [21], [22], [23], [24], [25], and we have previously found that the proportion of polycomb targeted miRNA genes is about three fold greater than protein coding genes [5]. Therefore, we examined whether polycomb targeted miRNAs are more likely to be targets of aberrant DNA methylation in breast cancer. To this end we integrated our H3K27me3 data from HMEC [5] with publicly available H3K27me3 domain data from embryonic stem cells (ESC) from two independent studies [26], [27]. ESC represent here universal ancestors of all cell types including HMEC and HMEC represent ancestors of breast carcinoma cells. We found a significant overlap between miRNA promoters occupied by the polycomb specific H3K27me3 mark in either ESC or HMEC and those aberrantly methylated in cancer cells. The comparisons show that miRNA promoters hypermethylated in cancer cell lines are significantly enriched in regions targeted by polycomb in both ESC and HMEC (Figure 5). Similar results were obtained when the analysis was restricted to non-CTS miRNA promoters and the hypermethylation occurring in tumor tissue samples (Figure S1). These observations provide additional evidence for the link between polycomb repression and aberrant DNA methylation in cancer. The predisposition of polycomb target genes to aberrant DNA methylation in cancer, together with the higher frequency of polycomb targets among miRNA genes, might explain the higher than expected proportion of miRNA genes with aberrant DNA methylation observed in breast cancer cells.

**Figure 6:**
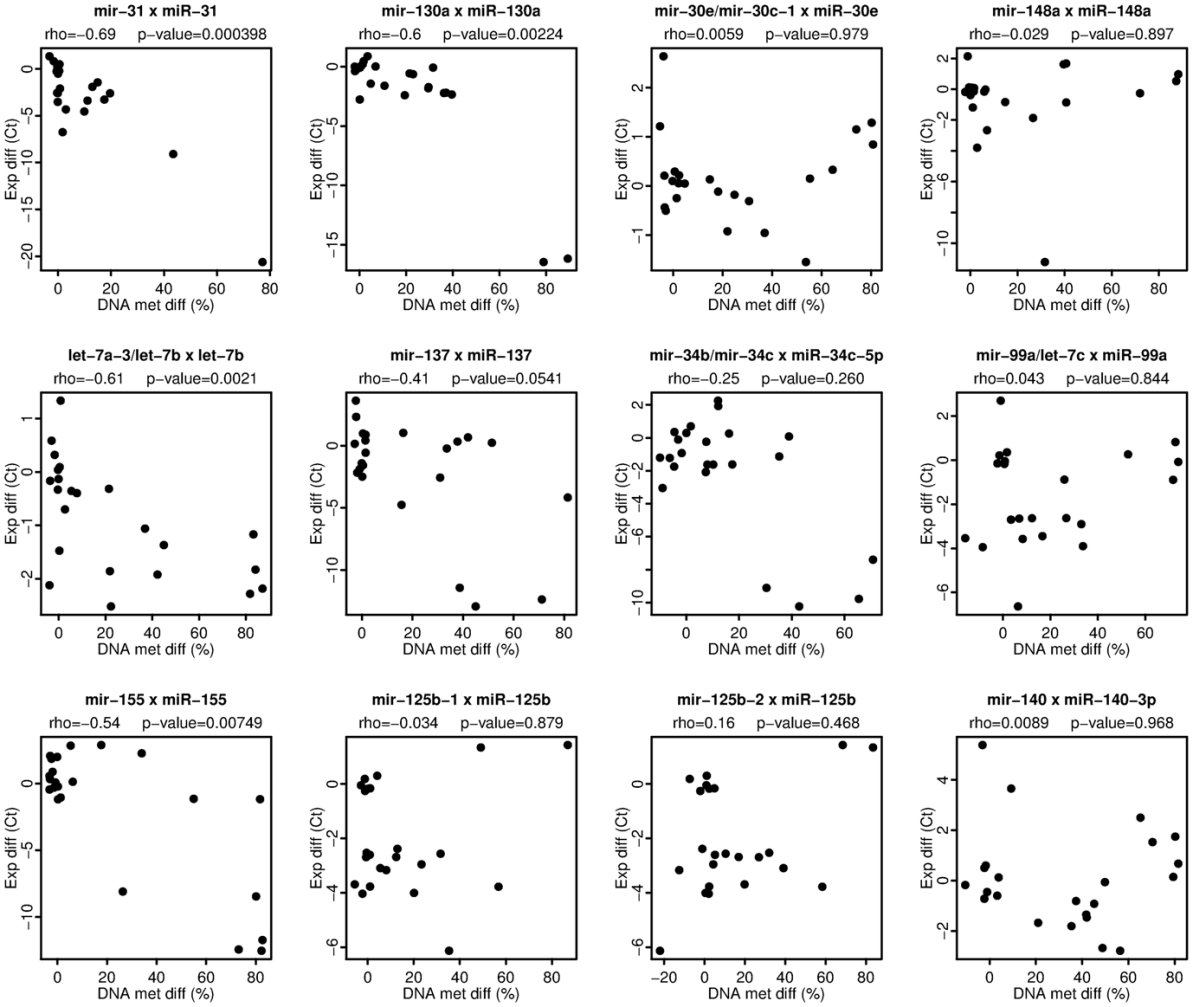
Aberrant DNA methylation of miRNA gene promoters in cancer is linked to silencing of miRNA expression. Each plot displays the relation between DNA methylation differences and miRNA expression differences from control in individual samples including breast cancer cell lines, breast tumor specimens, three normal HMEC, and normal breast tissue samples. The x-axis shows the difference in DNA methylation (%) between the sample and a normal reference for the whole MassARRAY amplicon. The y-axis shows the difference in miRNA expression (dCt) between the sample and a normal reference as determined by real-time PCR analysis. The names of miRNA genes/clusters analyzed by MassARRAY and the names of respective mature miRNA products detected by real-time PCR are displayed at the top of each plot. The Spearman correlation coefficient rho and the p-value (two-sided) of the correlation are displayed at the second line at the top of each plot.

Aberrant DNA methylation in cancer cells is often linked to gene silencing. We therefore analyzed the expression levels of the aberrantly methylated miRNA genes using quantitative real-time PCR. In general, negative or no correlation between miRNA expression and DNA methylation was observed (Figure 6), no positive correlations were found. Six out of twelve analyzed miRNA genes show a negative correlation between the level of DNA methylation determined by Sequenom MassARRAY and miRNA expression determined by real-time PCR. The negative correlation is significantly strong for four miRNAs (miR-31, miR-130a, let-7b, miR-155) and moderate or weak for miR-137 and miR-34c respectively (Figure 6). The other six miRNA genes did not display a significant relation between DNA methylation and miRNA expression level. This may be due to the tissue heterogeneity, where the presence of normal cells could obscure the detected miRNA level in tumor samples. In addition to DNA methylation, other factors like variable level of transcription factors in individual cancers may also be involved in variation of miRNA levels in these cases. Overall, these data confirm that the aberrant DNA methylation of miRNA gene promoters is linked to silencing of miRNA genes in cancer.

## Discussion

In this study, we sought to determine the DNA methylation status of miRNA promoters in breast cancer cells on the whole genome scale. To this end, we used 5-methylcytosine immunoprecipitation linked to a custom tiling microarray hybridization to analyze DNA methylation level in a variety of *in vitro* and *in vivo* representations of normal and diseased mammary cells. We found a large portion of analyzed miRNA promoters to be hypermethylated in breast cancer cell lines, and the same promoters were targeted in breast tumor tissues, although less frequently. The hypermethylation of selected miRNA promoters was verified using MassARRAY technology. MassARRAY DNA methylation data combined with miRNA expression data revealed that in six out of twelve cases the hypermethylation of miRNA promoters was linked to silencing of miRNA expression; in the other six cases DNA methylation did not correlate with the miRNA level. Overall, our study has shown aberrant DNA methylation of a large portion of miRNA genes leading frequently to silencing of miRNA expression in breast cancer. Since miRNAs play an important role in the regulation and maintenance of cell identity, our observations suggest that the deregulation of miRNA expression due to aberrant DNA methylation is involved in human breast carcinogenesis.

Almost one third of miRNA promoters active in normal mammary cells were found to contain hypermethylated DNA regions in breast cancer cells; this high proportion is several fold greater than usually observed for protein coding genes [19], [20]. However, miRNAs serve in general as regulators, being essential for proper regulation of gene expression in individual cells within an organism, but not necessarily critical for the survival of individual cells. Thus expression of most miRNAs is likely dispensable for cell survival, which may account for the frequent deregulation of miRNA expression in cancer cells that have escaped normal organismal level regulation. miRNA expression varies among normal cell types with most miRNAs having some level of CTS. Many of the CTS miRNA genes are physiologically repressed in non-expressing cell types by DNA methylation or, more often, by polycomb specific H3K27me3 [5]. The proportion of polycomb targets among miRNA genes is therefore high, about three fold higher than in protein coding genes [5]. Aberrant DNA hypermethylation preferentially targets polycomb repressed protein coding genes [21], [22], [23], [24], [25] and we found similar enrichment for polycomb targets among aberrantly methylated miRNA genes. The higher portion of polycomb targets among miRNA genes may therefore explain the high proportion of miRNA promoters with aberrant DNA methylation in cancer. However, it is possible that no direct functional link between aberrant DNA methylation and polycomb repression exists and both phenomena may significantly overlap just due to both affecting the genes that are not vital for survival of individual cells. Such genes are unmethylated in normal cells and repressed by polycomb in most normal cell types. If they get randomly hypermethylated during malignant transformation, their repression does not lead to the cell death. Some of these genes, e.g. CDKN2A (p16), have tumor suppressive functions and are inducible in normal cells, although repressed by polycomb. In case these genes gain aberrant DNA methylation, their inducibility is lost; the cells gain selective advantage and aberrant DNA methylation may thus contribute to the rise of the malignant phenotype.

We have previously found a significant portion of miRNAs expressed in normal breast cells to be cell-type specific [5] and we found here that more than half of these miRNA genes are hypermethylated in breast cancer cell lines (Figure 1E, Table 1). However, analyzing these types of miRNAs in heterogeneous tissues is problematic. We have previously shown that some of the CTS miRNA genes are differentially methylated in non-expressing normal cell types [4], [5]. Therefore, tissue samples with a variable proportion of expressing and non-expressing normal cell types may show differences in DNA methylation for these CTS miRNA genes, although these differences do not have a pathological origin. Normal breast tissue contains, in addition to epithelial cells, a substantial fraction of mesenchymal cells, particularly fibroblasts. The breast tumor tissue, on the other hand, contains a fraction of both normal epithelial and normal mesenchymal cells in addition to the carcinoma cells of epithelial origin. When we compared tumor to non-tumor tissue, we found mostly hypomethylation of HMEC specific miRNA genes including mir-205 and mir−200c/mir−141, that are hypermethylated in cancer cell lines, and we found only hypermethylation of HMF specific miRNAs (Table S1), most likely due to the lower level of normal mesenchymal cells in tumor versus non-tumor breast tissue. Non CTS miRNA genes, on the other hand, have no (or very little) physiological differences in DNA methylation between epithelial and mesenchymal cells and the results from tumor tissue were highly concordant with the results from pure cancer cell lines (Figure 2).

The incidence and magnitude of miRNA promoter hypermethylation found in breast tumor tissues was lower than in breast cancer cell lines (Figure 3). Two factors likely contribute to this observation. First, tumor tissues contain, in addition to tumor cells, a variable proportion of normal cells of various cell types that do not bear the hypermethylation and effectively dilute the sample making detection more difficult. Second, the tumor cells found within the tumor tissues are heterogeneous populations under evolution [28] with potentially different degree of aberrancy, and therefore all the tumor cells within one tumor may not bear a particular hypermethylation. While each tumor may contain many more hypermethylated regions, most of these regions might be present in only a small fraction of the tumor cells and therefore undetectable. None of the two above mentioned factors apply for the cancer cell lines, that are mostly uniform populations of clonal origin, and therefore the DNA methylation level detected here may be close to 100%.

Our study has shown that in six out of twelve miRNA genes analyzed in more detail the aberrant DNA methylation was linked to decreased levels of miRNAs in cancer. In addition to mir-137 and mir-34b/mir-34c genes, that were previously reported to be silenced by DNA methylation in various cancers [10], [11], [12], [29], [30], we found mir-31, mir-130a, let-7a-3/let-7b, and mir-155 to be silenced by aberrant DNA methylation in breast cancer. Interestingly, the mir-31 gene, located next to CDKN2A (p16) on chromosome 9, is homozygously deleted in MDA-MB-231 breast cancer cell line (data not shown and [31]), indicating that both, epigenetic and genetic mechanisms are involved in inactivation of miR-31 in various tumors.

Overall, our study reports the first systematic identification of aberrant DNA methylation of miRNA gene promoters in breast cancer. Aberrant DNA methylation found in breast cancer cell lines is also detectable in *in vivo* breast tumor tissues and might be therefore clinically relevant. The miRNA targets of aberrant DNA methylation might be potentially used for diagnostic purposes. miRNA genes silenced by aberrant DNA methylation in cancer are potential targets for epigenetic drugs.

## Materials and Methods

### Cell Lines and Tumor Specimens

HMEC and HMF were previously described [32] and grown as described [5], [32]. Breast cancer cell lines MCF7, MDA-MB-157, MDA-MB-231, MDA-MB-453, MDA-MB-468, BT549 were obtained from American Type Culture Collection (ATCC), and UACC1179 was described previously [33]. Cell line identity was assured by DNA fingerprinting using single tandem repeats. The cells were cultured as previously described [34], [35]. Flash-frozen breast specimens derived from non-tumor or cancerous breast tissue were obtained from patients who underwent surgery for breast cancer, either lumpectomy or mastectomy, at the University Medical Center in Tucson from 2003 to 2005. The study was approved by the University of Arizona Institutional Review Board and all patients signed surgical and clinical research consents for tissue collection in accordance with the University of Arizona Institutional Review Board and Health Insurance Portability and Accountability Act regulations. The tumor tissue processing and a partial molecular characterization of these samples have been previously reported [19], [36], [37]. An additional 14 breast tumor DNA samples were purchased from OriGene Technologies (Rockville, MD, USA).

### Nucleic Acid Extraction

Genomic DNA was extracted using the DNeasy Blood and Tissue Kit (Qiagen, Valencia, CA, USA) according to manufacturer protocol and quantified spectrophotometrically. Total RNA was extracted using the miRNeasy kit (Qiagen) according to manufacturer protocol except that Trizol (Invitrogen, Carlsbad, CA, USA) was used instead of Qiazol for the cell lysis. Each tissue sample was ground frozen in liquid nitrogen and resulting powder was then split for RNA and DNA extractions, respectively, to achieve homogenous representation of tissue components in both DNA and RNA samples.

### Microarray Analysis of DNA Methylation

The design of the microarray used in this study and MeDIP chip procedure was previously described [5]. The microarrays were manufactured by Agilent Technologies (Santa Clara, CA, USA). Microarray data (*.gpr files) were imported to R [38] using the limma package. Individual channels were first spatially normalized within arrays using ma2D function from the package marray and then loess normalized between arrays using the function normalize.loess from package affy. The RG object was transformed to an MA object and M values were again loess normalized between arrays. M values (log2 ratios of immunoprecipitated and input channel) were used for further analysis as a measure of enrichment of a region centered on individual probes. Data were analyzed in a sliding window of 1200 bp, the step was one probe. Differentially methylated regions (DMRs) were defined as regions of at least three consecutive probes less than 600 bp apart where the mean difference of ratios was at least 1.5 fold and such p-value cut was used so that the false discovery rate (FDR) stay≤5%. The FDR was determined by analysis of permutated data. miRNA promoters were considered to contain DMRs if there was a DMR overlapping within a 2kb region centered on the predicted TSS. The microarray data have been submitted to the NCBI Gene Expression Omnibus (GEO) (http://www.ncbi.nlm.nih.gov/geo/) under accession no GSE38254.

### Public Data

H3K27me3 domain data from H1 ESC were downloaded from two sources: supplemental data from [26] and the data track from UCSC [27]. To determine more stringent H3K27me3 domains, the overlap of the data from these two sources was used in our analysis.

### DNA Methylation Analysis by MassARRAY

DNA methylation analysis by Sequenom MassARRAY (Sequenom, San Diego, CA,USA) was performed as described [37]. MassARRAY amplicons 350–550 bp in length were targeted to the part of the 2 kb region centered on TSS, where the DMRs were present in most samples according to the microarray data. Primer sequences and positions are listed in Table S2. Oligonucleotides used for MassARRAY analysis were ordered from Integrated DNA Technologies (Coralville, IA, USA).

### Real-time PCR Analysis of miRNA Expression

Expression of microRNAs was performed in triplicate using EXIQON miRCURY LNA™ Universal RT kit and EXIQON miRCURY LNA™ Universal RT microRNA PCR, Pick-&-Mix, Ready-to-use Panels (EXIQON, Woburn, MA, USA). Real-time PCR was conducted on an ABI Prism 7500 Sequence Detection System (Applied Biosystems, Foster City, CA, USA) using PerfeCta SYBR Green SuperMix, Low ROX (Quanta Biosciences, Gaithersburg, MD, USA) with a 95°C denaturation for 3 minutes followed by 40 cycles of 95°C for 15 seconds and 60°C for 45 seconds. Differences in expression were determined using the comparative Ct method described in the ABI user manual. SNORD38B and SNORD49A expression was used for normalization of miRNA expression.

### Statistical Analysis

The significance of promoter region overlaps was calculated in R using hypergeometric test as described previously [39]. Spearman correlation coefficient rho between differences in DNA methylation and miRNA expression was calculated using function cor.test in R.

## Supporting Information

Figure S1 **MicroRNA promoters hypermethylated in cancer are frequently occupied by polycomb specific H3K27me3 in normal cells.** Left, Venn diagram showing the overlap between the non-CTS miRNA promoters occupied by H3K27me3 in ESC and those hypermethylated in tumor tissue samples (TT). Right, Venn diagram showing the overlap between the non-CTS miRNA promoters occupied by H3K27me3 in HMEC and those hypermethylated in TT. Both overlaps are highly significant (hypergeometric test).
(PDF)

Table S1 **DNA methylation status for all analyzed miRNA promoters.** The table shows the presence or absence of DMRs for individual miRNA promoters for groups of samples and individual samples. The first column shows the names of individual miRNA genes: non CTS genes have yellow background, HMEC-specific green background and HMF-specific red background. The second column shows the expression ratio of these miRNA between two normal mammary cell types: HMEC and HMF. The next three columns indicate whether the promoter region is occupied by polycomb specific H3K27me3 in three normal cell types: H1 ESC, HMEC and HMF. Then folows the DNA methylation data for all cancer cell lines compared to HMEC, all tumor tissue compared to nontumor tissue and for HMF compared to HMEC. The last section shows hypermethylated DMRs for individual cancer cell lines relative to HMEC and individual tumor tissue samples relative to nontumor tissue. The data are color coded, hypermethylated DMRs are indicated by brown color, hypomethylated DMRs by green color. The area of cell type specific miRNA promoters for tissue samples is shaded grey since this part of data is prone to missinterpretation.
(XLS)

Table S2 **Positions of MassARRAY amplicons in hg18 and sequences of MassARRAY primers used for DNA methylation analysis.**
(PDF)
