# Supplementary material for: miRNA Gene Promoters Are Frequent Targets of Aberrant DNA Methylation in Human Breast Cancer
Source: PLoS One. 2013 Jan 16;8(1):e54398. doi: 10.1371/journal.pone.0054398 (PMC3547033; doi:10.1371/journal.pone.0054398)
Supplement: Figure S1 — MicroRNA promoters hypermethylated in cancer are frequently occupied by polycomb specific H3K27me3 in normal cells. Left, Venn diagram showing the overlap between the non-CTS miRNA promoters occupied by H3K27me3 in ESC and those hypermethylated in tumor tissue samples (TT). Right, Venn diagram showing the overlap between the non-CTS miRNA promoters occupied by H3K27me3 in HMEC and those hypermethylated in TT. Both overlaps are highly significant (hypergeometric test). (PDF) [file pone.0054398.s001.pdf]

Figure S1

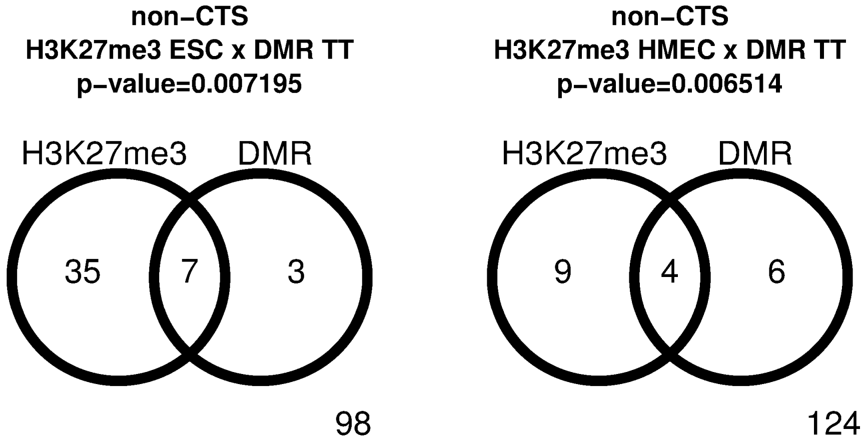

MicroRNA promoters hypermethylated in cancer are frequently occupied by polycomb specific H3K27me3 in normal cells. Left, Venn diagram showing the overlap between the non-CTS miRNA promoters occupied by H3K27me3 in ESC and those hypermethylated in tumor tissue samples (TT). Right, Venn diagram showing the overlap between the non-CTS miRNA promoters occupied by H3K27me3 in HMEC and those hypermethylated in TT. Both overlaps are highly significant (hypergeometric test).
