# Supplementary material for: miRNA Gene Promoters Are Frequent Targets of Aberrant DNA Methylation in Human Breast Cancer
Source: PLoS One. 2013 Jan 16;8(1):e54398. doi: 10.1371/journal.pone.0054398 (PMC3547033; doi:10.1371/journal.pone.0054398)
Supplement: Table S2 — Positions of MassARRAY amplicons in hg18 and sequences of MassARRAY primers used for DNA methylation analysis. (PDF) [file pone.0054398.s003.pdf]

Table S2

Positions of MassARRAY amplicons in hg18 and sequences of MassARRAY primers used for DNA methylation analysis

chr	start	end	amplicon name	10F primer sequence	T7R primer sequence
chr9	21,549,054	21,549,490	mir-31	aggaagagagTTTTTTTTAAGAAGGGAAAGTTTAGTTA	cagtaatacgactcactatagggagaaggctCAAATAAACTAAAAAAACCTTAATCCC
chr11	57,161,896	57,162,383	mir-130a	aggaagagagAGGTTTTTATTGGTTGTGGGTTAAT	cagtaatacgactcactatagggagaaggctTTCTAAAACTACCCTCTTCCTAACCA
chr22	44,856,162	44,856,743	let-7a-3/let7b	aggaagagagTGGGTGGTATGAATTTTTTAGTTTG	cagtaatacgactcactatagggagaaggctAAAATCCCTCAAAAACCTTTAAATC
chr1	98,284,168	98,284,631	mir-137	aggaagagagGTTTAGTGGTTAGTTGGTGGGTTG	cagtaatacgactcactatagggagaaggctATACCCAAAAAACCTTACCACATCT
chr21	25,855,810	25,856,217	mir-155	aggaagagagTGTTTGTTTTTGGAATTTATAGTTTGA	cagtaatacgactcactatagggagaaggctCCATTTCTTCCTCTCTTAAAAACCTA
chr16	68,516,150	68,516,613	mir-140	aggaagagagGGTTTAAGTGAGTTTTGGAAAAGGT	cagtaatacgactcactatagggagaaggctCAACTTCAAATTCAAACAAAAAAAA
chr1	40,947,229	40,947,768	mir-30e/mir-30c-1	aggaagagagTAGGAGATGTAGAAATATGAGTTTGGAA	cagtaatacgactcactatagggagaaggctAAACTCTAACAACCAATAAAAAATCCC
chr7	25,956,149	25,956,538	mir-148a	aggaagagagGTTTTTAGTTTTTTAGATGGGAAGGT	cagtaatacgactcactatagggagaaggctATCTTCCTCCAAAAATTTCTTCCTA
chr11	110,888,268	110,888,617	mir-34b/mir-34c	aggaagagagTTTTTTTTGGAGGTTTTTTAGGGAT	cagtaatacgactcactatagggagaaggctAAAAATACCAAACCTCCCCTTC
chr21	16,828,628	16,829,184	mir-99a/let-7c	aggaagagagTATTTTTGGTTGATGGGTAGAAAAA	cagtaatacgactcactatagggagaaggctCCAATCAAAAAAAACAAAAAAAACA
chr11	121,476,136	121,476,612	mir-125b-1	aggaagagagTATAAATGATTGAAGAGAAAATAGGAGGT	cagtaatacgactcactatagggagaaggctCTCTAAAAAAATTTCATACTCCCCC
chr21	16,882,554	16,882,936	mir-125b-2	aggaagagagTTGTTTTTTATTGATTGATTGGTTT	cagtaatacgactcactatagggagaaggctCCCTAAAAAAAATAAAAACATTTCC
